# Serum IgG-induced microglial activation enhances neuronal cytolysis via the NO/sGC/PKG pathway in children with opsoclonus-myoclonus syndrome and neuroblastoma

**DOI:** 10.1186/s12974-020-01839-9

**Published:** 2020-06-16

**Authors:** Xu Ding, Wei Yang, Qinghua Ren, Jiajian Hu, Shen Yang, Wei Han, Jing Wang, Xu Wang, Huanmin Wang

**Affiliations:** 1 Laboratory of Nutrition and Development, Beijing Pediatric Research Institute, Beijing Children’s Hospital, Capital Medical University, National Center for Children’s Health, No. 56 Nan-li-shi Road, Xi-Cheng District, Beijing, 100045 China; 2 Department of Surgical Oncology, Beijing Children’s Hospital, Capital Medical University, National Center for Children’s Health, Beijing, 100045 China; 3 Department of Neurobiology, School of Basic Medical Sciences, Beijing Institute for Brain Disorders, Capital Medical University, Beijing, 100069 China; 4 Department of Neurology, Beijing Children’s Hospital, Capital Medical University, National Center for Children’s Health, Beijing, 100045 China

**Keywords:** Opsoclonus-myoclonus syndrome, Neuroblastoma, Children, Microglial activation, Nitric oxide (NO), Soluble guanylyl cyclase (sGC), Protein kinase G (PKG)

## Abstract

**Background:**

Opsoclonus-myoclonus syndrome (OMS) is a rare neurological disease. Some children with OMS also have neuroblastoma (NB). We and others have previously documented that serum IgG from children with OMS and NB induces neuronal cytolysis and activates several signaling pathways. However, the mechanisms underlying OMS remain unclear. Here, we investigated whether nitric oxide (NO) from activated microglias and its cascade contribute to neuronal cytolysis in pediatric OMS.

**Methods:**

The activation of cultured cerebral cortical and cerebellar microglias incubated with sera or IgG isolated from sera of children with OMS and NB was measured by the expression of the activation marker, cytokines, and NO. Neuronal cytolysis was determined after exposing to IgG-treated microglia-conditioned media. Using inhibitors and activators, the effects of NO synthesis and its intracellular cascade, namely soluble guanylyl cyclase (sGC) and protein kinase G (PKG), on neuronal cytolysis were evaluated.

**Results:**

Incubation with sera or IgG from children with OMS and NB increased the activation of cerebral cortical and cerebellar microglias, but not the activation of astrocytes or the cytolysis of glial cells. Moreover, the cytolysis of neurons was elevated by conditioned media from microglias incubated with IgG from children with OMS and NB. Furthermore, the expression of NO, sGC, and PKG was increased. Neuronal cytolysis was relieved by the inhibitors of NO signaling, while neuronal cytolysis was exacerbated by the activators of NO signaling but not proinflammatory cytokines. The cytolysis of neurons was suppressed by pretreatment with the microglial inhibitor minocycline, a clinically tested drug. Finally, increased microglial activation did not depend on the Fab fragment of serum IgG.

**Conclusions:**

Serum IgG from children with OMS and NB potentiates microglial activation, which induces neuronal cytolysis through the NO/sGC/PKG pathway, suggesting an applicability of microglial inhibitor as a therapeutic candidate.

## Background

Opsoclonus-myoclonus syndrome (OMS) is a rare but devastating neurological disease, characterized by opsoclonus, myoclonus, and ataxia. Most patients suffer from persistent deficits in cognition, neurology, and behavior. Some children with OMS also have neuroblastoma (NB), although varied percentages have been reported [1–5]. Previously, we have documented that the insulin-like growth factor 1 (IGF-1)/phosphoinositide 3-kinase (PI3K) cascade is compensatively activated to alleviate neuronal cytolysis induced by serum IgG from children with OMS and NB [6], and others reported that the phosphorylation of extracellular signal-regulated kinase contributes to neuronal cytolysis in pediatric OMS [7]. Yet, the cellular and molecular mechanisms associated with neuronal cytolysis underlying pediatric OMS remain unclear.

Microglias are major immune effectors in the central nervous system (CNS) and have an important physiological function in inflammation [8]. Notably, patients with pediatric OMS exhibit increased expression of a microglial marker and proinflammatory cytokines in cerebrospinal fluid (CSF) and some children with OMS are post-infectious [9–11]. Furthermore, serum IgG or autoantibody existed in patients enhances microglial activation in Parkinson disease (PD) [12], amyotrophic lateral sclerosis (ALS) [13], and systemic lupus erythematosus (SLE) [14–16]. Thus, it is reasonable to hypothesize that serum IgG from children with OMS and NB may impact the activation of microglia.

Activated microglias release various neurotoxic molecules [8, 17], causing the loss of neurons in neurodegenerative diseases [18–20]. We and others have revealed the cytolysis of cultured cerebellar and cerebral neurons by sera or IgG from patients with pediatric OMS and NB [6, 7]. Additionally, activated microglias can synthesize and release nitric oxide (NO), which can activate soluble guanylyl cyclase (sGC) and protein kinase G (PKG) in neurons, thereby contributing to neuronal death by mitochondrial dysfunction [21, 22] and increased neuronal susceptibility to mitochondrial dysfunction [23]. However, whether the cytolysis of neurons can be induced by activated microglias in children with OMS and NB via the NO/sGC/PKG cascade is still unknown.

Here, we found that sera or serum IgG from children with OMS and NB induces the activation of cultured cerebral cortical and cerebellar microglias, but not astrocytes. The cytolysis of neurons is exerted by conditioned media from microglias treated with IgG from children with OMS and NB. Furthermore, the NO/sGC/PKG pathway contributes to neuronal cytolysis induced by conditioned media, and neuronal cytolysis can be almost completely suppressed by pretreatment with the microglial inhibitor minocycline, a clinically tested drug. Finally, increased microglial activation may depend on the Fc fragment of serum IgG rather than the Fab fragment. Our results suggest that serum IgG from children with OMS and NB increases the activation of cultured microglias, leading to the upregulation of NO, which subsequently activates sGC and PKG in neurons, thereby inducing neuronal cytolysis. These results also suggest that the microglial inhibitor, such as minocycline, may serve as a plausible therapeutic candidate.

## Methods

### Subjects

#### Enrolled participants

This project was reviewed and approved by the Ethics Committees of Beijing Children’s Hospital, Capital Medical University (No. IEC-C-028-A10-V.05). Parents of participants signed written informed consent. Children were enrolled between January 2015 and December 2019 from Beijing Children’s Hospital using internationally accepted diagnostic criteria for OMS [24] and they were Han Chinese. Ten children with OMS and NB were collected (OMS + NB). Control groups were 20 children with NB without OMS (NB), 10 age- and sex-matched healthy children (healthy control), 10 children with juvenile idiopathic arthritis (JIA) to reveal whether the effects on microglias are common to all IgG-related diseases, and 6 children with anti-*N*-methyl-*d*-aspartate receptor (NMDAR) encephalitis to investigate whether the effects on microglias are common to all autoantibody-mediated disorders of the CNS. Two individuals in the OMS + NB group, 3 individuals in the NB group, and all the individuals in the anti-NMDAR encephalitis were newly collected, while the rest subjects were previously described in our another study [6].

#### Clinical information

Demographic and clinical information is shown in Table 1. The age and gender of 5 groups, as well as the tumor stage of the NB and OMS + NB groups, were not statistically different. The age of subjects was at the time of our study, and thus, it may be older than the age of onset. Although a higher female sex ratio of approximately 1.2 was already evident in toddlers with OMS [2–4], only 10 children with OMS and NB in our study may not enough to reflect the gender difference, and other literatures with a small sample size also showed much lower (2 females/3 males) [25] or higher ratios (9 females/2males, 11 females/4 males) [7, 26]. Moreover, the age and gender of subjects may be affected by whether timely visiting to hospitals, misdiagnosis as other diseases before, genetic background, or willingness to enroll in the study. All the children in the JIA group had systemic-onset JIA. Electroencephalogram (EEG) results of children with OMS were clinically normal, while EEG results of all the children with anti-NMDAR encephalitis were abnormal. All the blood samples of the NB and OMS + NB groups were recruited before surgery for NB, and most of the blood samples were recruited before any treatment. After collection, sera were stored at − 80 °C.

**Table 1:** Demographic and clinical data

	*n*	Age enrolled in our study (month)	Gender (female/male)	Tumor stage	Treatment before serum collection
Healthy children	10	41.9 ± 1.7	5/5	–	–
NB	20	44.5 ± 6.4	9/11	I: 4 II: 9 III: 6 IV: 1	13 children: no treatment 7 children: chemotherapy, receiving vincristine, cyclophosphamide, adriamycin, etoposide, and cisplatin
OMS + NB	10	35.6 ± 9.9	5/5	I: 3 II: 4 III: 3 IV: 0	8 children: no treatment 1 child: steroid 1 child: intravenous immune globulin
Juvenile idiopathic arthritis (JIA)	10	40.8 ± 10.5	6/4	–	6 children: no treatment 3 children: nonsteroidal anti-inflammatory drugs 1 child: received steroid
Anti-NDMAR encephalitis	6	54.3 ± 9.9	4/2	–	2 children: no treatment 4 children: steroid and intravenous immune globulin

According to the criteria for evaluating OMS [1], the degree of ataxia, opsoclonus, ataxia/gait, ataxia/stance, and mood/sleep disturbance of patients with OMS was graded from 0 to 3 and summarized into OMS score in Table 2. The frequency of symptoms was similar to a previous report [4].

**Table 2:** OMS scores of children with OMS and NB at the time of the blood draw

Symptom	Number of children				%
	Grade 0	Grade 1	Grade 2	Grade 3	
Opsoclonus	1	4	1	4	90
Myoclonus	3	5	2	0	70
Ataxia/gait	4	4	2	0	60
Ataxia/stance	6	4	0	0	40
Mood/sleep disturbance	5	5	0	0	50

### Purification of IgG and preparation of the Fab fragment

As we previously used [6], 100 μl of protein G agaroses (Thermo Fisher Scientific, Sunnyvale, CA, USA) were applied to sera. The IgG-free fraction of sera in supernatants was collected after centrifugation at 10000 g for 10 min. The IgG fraction of sera was eluted by 0.1 M glycine-HCl (pH 2.7) after washing with 0.01 M phosphate buffer saline (PBS), and then neutralization buffer was added. The BCA assay (Pierce, Rockford, IL, USA) was applied to determine IgG concentration.

The Fab fragment of IgG was prepared by enzymatic digestion [16]. Pepsin (Sigma-Aldrich, St. Louis, MO, USA) was mixed with IgG at a ratio of 1:20 and then incubated at 37 °C for 6 h. The pH of solution was adjusted to 7.4 to stop the digestion. HiTrap Protein G HP columns (GE Healthcare, Freiburg, Germany) and Amicon Ultra-15 centrifugal filters (Merck Millipore, Billerica, MA, USA) were used to obtain the Fab fragment.

### Primary cultures of microglias or astrocytes separately

Primary cultures of microglias [27] or astrocytes [28] were prepared as previously described. Sprague Dawley rats at postnatal day 1 were used, which were provided by the Department of Experimental Animal Sciences, Capital Medical University (Beijing, China). The cerebral cortex and cerebellum were cut and digested in 0.1% trypsin at 37 °C for 20 min, and then, tissues were triturated gently. After filtration through a 30-μm cell drainer (BD Biosciences Discovery Labware, Bedford, MA, USA), cell suspensions were centrifuged at 1500 rpm for 5 min and resuspended in Dulbecco’s modified Eagle’s medium (Life Technologies, Rockville, MD, USA) with 10% inactivated low-endotoxin fetal bovine serum.

For primary culture of microglias, mixed glial cultures were shaken at 200 rpm for 2 h after seeding onto lysine-coated dishes for 7 days. After pelleting, floating cells were subcultured at 3.0 × 10^5^ cells/2000 μl medium/well in 6-well plates or at 1.0 × 10^4^ cells/200 μl medium/well in 96-well plates. One day after subculture, the medium was fully replaced by macrophage serum-free medium. Cells were used 2 days later.

For primary culture of astrocytes, mixed glial cultures were shaken at 200 rpm for 2 h after seeding onto dishes for 10 days. Adherent cells were trypsinized (0.05%) and subcultured at 3.0 × 10^5^ cells/2000 μl medium/well in 6-well plates with glial culture medium. One day after subculture, plates were manually shaken for 1 min and the medium was fully replaced with glial culture medium. Cells were used 7–10 days later.

### Primary culture of neurons

Primary culture of neurons was carried out according to methods previously described [6]. The cerebral cortex and cerebellum of Sprague Dawley rats (16 to 18 days old) were used. Notably, 24 h after seeding neurons, cytosine arabinoside at 10 μM was added to suppress glial proliferation and prepare neuron-enriched cultures.

### Treatment of sera, IgG, or chemicals

#### Microglias or astrocytes incubated with sera or IgG

As we used previously [6], the culture medium of microglias or astrocytes was replaced with a fresh medium containing sera or IgG from children (6-well plates in a 2000-μl volume: 40 μl sera of children, 1:50 diluted; 100–200 μg IgG; 1000–1400 μg the IgG-free fraction; 96-well plates in a 200-μl volume: 4 μl sera of children, 1:50 diluted; 10–20 μg IgG). Microglias or astrocytes were incubated with sera or IgG for 48 h before performing further assays, such as ELISA, or getting conditioned media. To explore whether increased microglial activation is simply induced by higher concentration of IgG, commercially available human IgG (Sigma-Aldrich) was dissolved in normal saline and was added into each well with the medium at the final concentration of 0.1 μg/μl and the highest concentration in the OMS + NB group. Each serum or IgG of an individual parallelly treated 3 wells of glial cells. Cluster of differentiation 11b (CD11b) is a marker of microglial activation. Microglias to examine the expression of CD11b and conditioned media to detect cytokines or neuronal cytolysis were got from the same well.

#### Neurons incubated with conditioned media

In order to avoid the direct effects of serum IgG on neurons [6], instead of co-culture of microglias and neurons and treating both kinds of cells with IgG at the same time, here we cultured microglias or neurons separately. IgG from children was added into the culture media of microglias to collect conditioned media, and then, conditioned media were filtered with protein G agarose to get the IgG-free fraction. Finally, the culture media of neurons were replaced with IgG-free conditioned media from microglias. This method has been used before [29].

#### Microglias or neurons treated with chemicals

To investigate the role of NO and its intracellular cascade in neuronal cytolysis, the NO synthesis inhibitor 7-nitroindazole (7-NINA) was added into the media of microglias 30 min before treatment with the IgG fraction, whereas the sGC inhibitor 1H-1,2,4 oxadiazolo-4,3-a quinoxalin-1-one (ODQ) or the PKG inhibitor Rp-8Br-PET-cGMP (the inhibitor of both PKG type I and type II) (all from Tocris Biosciences, Bristol, UK) was added into the culture media of neurons 30 min before the replacement of conditioned media from microglias. While 7-NINA or Rp-8Br-PET-cGMP was dissolved in normal saline and added into the medium to make a final concentration of 10 μM or 1 μM, ODQ was dissolved in 5% dimethyl sulfoxide (DMSO, Sigma-Aldrich) at a final concentration of 10 μM.

Moreover, pharmacological activators of the NO signaling pathway were also tested. The NO-donor *S*-nitroso-*N*-acetylpenicillamine (SNAP) was added into the media of microglias 30 min before treatment of IgG from children, while the sGC activator 3-5-hydroxymethyl-2-furyl-1-benzyl-indazole (YC-1) or the PKG activator 8Br-cGMP (the activator of both PKG type I and type II) (all from Tocris Biosciences) was added into the culture media of neurons 30 min before the replacement of IgG-treated microglia conditioned media. Whereas SNAP or YC-1 was dissolved in 5% DMSO and added into the medium at 100 μM or 1 μM, 8Br-cGMP was dissolved in normal saline at a final concentration of 1 μM. The final concentration of DMSO was 0.01%. Additionally, minocycline (Sigma-Aldrich), an inhibitor of microglial activation, was given 30 min before SNAP or IgG from children. Minocycline was dissolved in normal saline and added into the medium at 20 μM.

Recombinant IL-1β, IL-6, TNF-α, or MCP-1 (all from R & D Systems, Minneapolis, MN, USA) was used 30 min before the replacement of neuronal medium. Cytokines were dissolved in 0.01 M PBS containing 0.1% bovine serum albumin (BSA) and were added into the culture medium of neurons at 100 pg/ml, 100 pg/ml, 80 ng/ml, and 20 ng/ml, respectively. The concentrations of inhibitors, activators, and recombinant cytokines were selected based on previous studies [23, 30].

To investigate the role of IGF/PI3K signaling in neuronal cytolysis, recombinant IGF-1 (R & D Systems) was added into the culture media of neurons 30 min before the replacement of conditioned media from microglias. IGF-1 was dissolved in 0.01 M PBS and was added at the final concentration of 10 nM. To detect the effects of PI3K on neuronal cytolysis, the PI3K inhibitor LY294002 (Sigma-Aldrich) was added into the culture media of neurons 30 min before IGF-1. LY294002 was dissolved in 5% DMSO and was added at the final concentration of 20 μM.

### Enzyme-linked immunosorbent assay (ELISA)

Cultured microglias, astrocytes, or neurons were lysed in RIPA buffer (KeyGen Biotechnology, Nanjing, China) after washing by PBS. Commercially available ELISA kits were applied to explore the expression of CD11b (Cloud-Clone, Houston, TX, USA) in microglias, glial fibrillary acidic protein (GFAP) (Abcam, Cambridge, MA, USA) in astrocytes, and PI3K (CUSABIO, Wuhan, China) [6] in neurons. The concentrations of CD11b, GFAP, and PI3K were normalized to the amount of total protein, which was detected at the same time as ELISA using the BCA protein assay kit.

Moreover, the culture medium of microglias was used to assess the concentrations of proinflammatory cytokines IL-1β (Bio-Swamp, Wuhan, China), IL-6 (Promocell, Heidelberg, Germany), TNF-α (Abcam), and MCP-1 (Abcam) by commercially available ELISA kits following manufacturer’s instructions.

### Nitrite assay

To calculate the concentration of NO in the culture medium of microglias, nitrite was measured, which is a product resulting from the reaction of NO with molecular oxygen. Briefly, 50 μl of cell culture media and an equal volume of Griess reagent (0.1% naphtyletylenediamine dihydrochloride, 1% sulphanilamide, 2.5% phosphoric acid, Sigma-Aldrich) were added in 96-well plates. The absorbance at 550 nm was determined, and standard curves with sodium nitrite (NaNO_2_, Sigma-Aldrich) were generated [27].

### Cytolysis assay

Cytoplasmic lactate dehydrogenase (LDH), an indicator of plasma membrane-damaged cells, was measured by the cytotoxicity detection kit (Roche, Indianapolis, IN, USA) [6]. Cytolysis of neurons or microglias was detected using the same method. Briefly, 100 μl of culture supernatants of neurons or microglias was mixed with an equal volume of reaction mixture followed by incubation for 30 min. LDH was detected by the absorbance at 490 nm. Maximum LDH release was induced by 1% Triton X-100, while the background control was untreated neurons or microglias. The percentage of specific cytolysis was determined using the following formula: [(experimental value-background control)/(maximum value-background control)] × 100.

### Western blot

To assess the protein expression of CD11b in the cell membrane, the cell membrane fraction of microglias was isolated using the plasma membrane protein extraction kit (Abcam). Protein concentration was determined by the BCA protein assay kit. After gel electrophoresis, proteins were transferred to PVDF membranes. PVDF membranes with the cell membrane fraction were rinsed briefly in distilled water and stained with Ponceau S solution (Po-S) (0.5 [w/v] in 1% [v/v] acetic acid) for 2 min, rinsed in distilled water to remove excess stain, and imaged. Protein levels were normalized to Po-S as a loading control (100–140 kDa) [31]. After blocking, rabbit anti-CD11b antibody (127 kDa, 1:1000, ab133357, Abcam) was incubated at 4 °C overnight. Following washing, PVDF membranes were then incubated for 1 h at room temperature with HRP-conjugated secondary antibody. Finally, an enzymatic chemiluminescence kit (Thermo Fisher Scientific) was used to visualize protein bands, and the intensity of protein bands was quantified using Quantity One software (Bio-Rad, Hercules, CA, USA).

To assess the protein expression of sGC or PKG in whole-cell lysates, neurons were homogenized in RIPA buffer. PVDF membranes with whole-cell lysates were blocked before incubating with rabbit anti-sGC β1 subunit antibody (70 kDa, 1:1000, G4405, Sigma-Aldrich), rabbit anti-PKG type I antibody (78 kDa, 1:1000, 3248, Cell Signaling Technology, Danvers, MA, USA), rabbit anti-PKG type II antibody (87 kDa, 1:1000, SAB4502387, Sigma-Aldrich), or mouse anti-glyceraldehyde 3-phosphate dehydrogenase (GAPDH) antibody (40 kDa, 1:5000, ab8245, Abcam). GAPDH was used as a loading control. Other steps were the same as described above.

### Statistical analyses

All the data were expressed as mean ± SEM. Statistical analyses were carried out using GraphPad Prism version 6.0 software for Windows (GraphPad Software Inc., San Diego, CA, USA). One-way analysis of variance (ANOVA) followed by Dunnett’s multiple comparison test was used for multiple analyzation. The statistically significant level was considered at *p <* 0.05.

## Results

### The activation of cerebral cortical and cerebellar microglias is increased by sera or IgG from children with OMS and NB

Besides the cerebellum, emerging evidence has shown that the cerebral cortex has structural and functional changes in OMS patients. First, most OMS patients have neurological handicaps in cerebral functions, such as deficits in attention, memory, and language [4, 32]. Second, brain imaging of OMS patients shows changes in the cerebrum. Cerebral cortical thickness is reduced across the motor and visual areas in patients with pediatric OMS [33]. A patient with OMS reveals significant nodular enhancing lesions at gray–white junction of bilateral cerebral hemispheres by magnetic resonance imaging [34]. Another patient shows decreased metabolism in the bilateral occipital lobes and increased functional connectivity, including the primary- and motion-sensitive visual cortex [35]. Therefore, both cerebral cortical and cerebellar microglias were exposed to sera or the IgG fraction from children with OMS and NB.

**Fig. 1:**
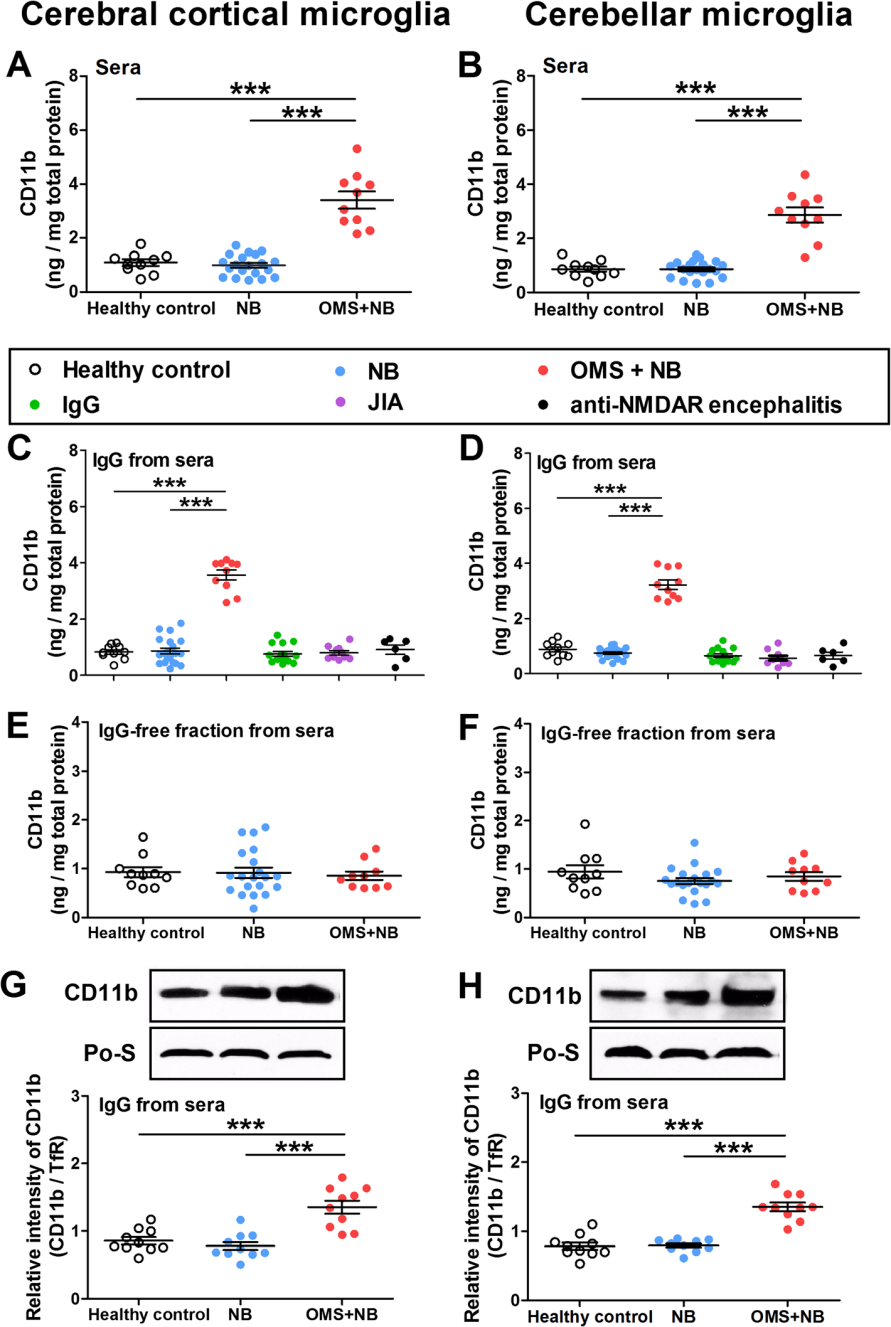
CD11b expression in cerebral cortical and cerebellar microglias incubated with OMS + NB sera or IgG. Note that the concentration of CD11b, a marker of microglial activation, was upregulated in cerebral cortical microglias (**a**) and cerebellar microglias (**b**) incubated with sera from children with OMS and NB (OMS + NB) compared with those from children with NB or healthy children. The IgG fraction (**c**, **d**) of the OMS + NB group also elevated the level of CD11b, but not the IgG-free fraction (**e**, **f**). No alteration was observed in the expression of CD11b after treatment with commercially available human IgG, the IgG fraction from children with juvenile idiopathic arthritis (JIA) or anti-NMDAR encephalitis (**c**, **d**). The expression of CD11b in the cell membrane of microglias was upregulated by IgG of the OMS + NB group (**g**, **h**). ^***^p < 0.001, one-way ANOVA, *n* = 10 (health control), *n* = 20 (NB), *n* = 10 (OMS + NB), *n* = 15 (IgG), *n* = 10 (JIA), *n* = 6 (anti-NMDAR encephalitis). *n* = 10/ group (**g**, **h**, Western blot)

The expression of CD11b, a marker of microglial activation, was upregulated in cerebral cortical microglias incubated with sera from children with OMS and NB (3.41 ± 0.32 ng/mg total protein OMS + NB, 0.99 ± 0.09 ng/mg total protein NB, 1.09 ± 0.12 ng/mg total protein healthy control, *p* < 0.001 vs NB, *p* < 0.001 vs healthy control), whereas CD11b concentration was not statistically changed by sera of children with only NB at least under our experimental conditions (Fig. 1a). Moreover, IgG isolated from sera upregulated CD11b expression from 0.91 ± 0.10 ng/mg total protein in the NB group and 0.88 ± 0.07 ng/mg total protein in the healthy control group to 2.95 ± 0.23 ng/mg total protein in the OMS + NB group (*p* < 0.001 vs NB, *p* < 0.001 vs healthy control, Fig. 1c). With respect to cerebellar microglias, the concentration of CD11b was also increased after incubation with sera or IgG from the OMS + NB group compared with those from the NB or healthy control group (Fig. 1b, d). However, neither commercially available human IgG nor IgG from children with JIA or anti-NMDAR encephalitis had a significant impact on the concentration of CD11b (Fig. 1c, d), suggesting that upregulation of CD11b induced by serum IgG from children with OMS and NB is not simply induced by increased dose of IgG, is not common to all diseases with IgG, and is not common to all autoantibody-mediated disorders of the CNS. In addition, no alteration of CD11b concentration was observed after treatment with the IgG-free fraction (Fig. 1e, f), which suggested that the upregulation of CD11b induced by sera mainly depends on the IgG fraction. Consistently, the protein expression of CD11b in the cell membrane fraction was increased in the cerebral cortical and cerebellar microglias incubated with OMS + NB IgG (Fig. 1 g, h).

**Fig. 2:**
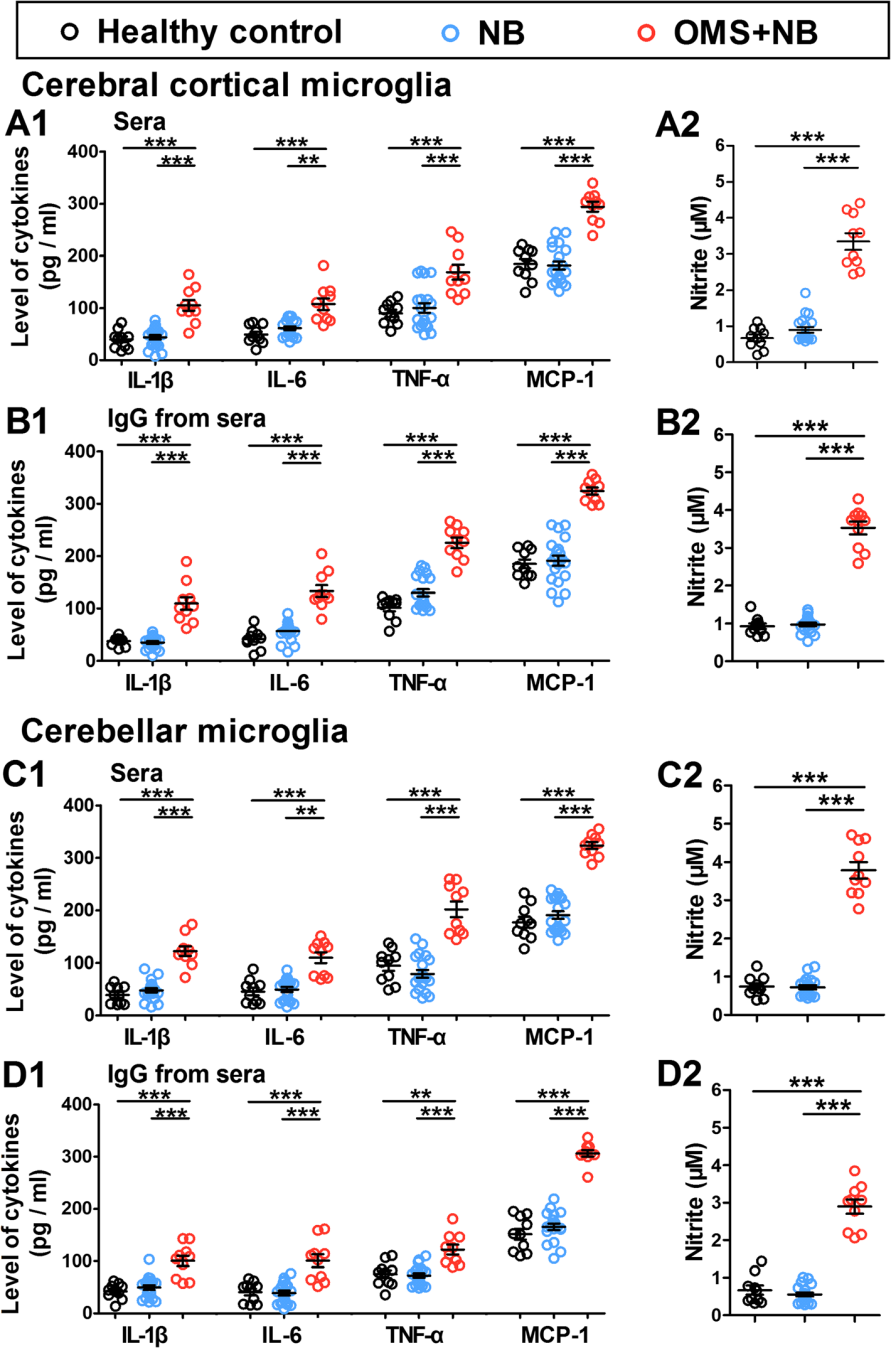
Proinflammatory cytokines and NO levels in the medium of cerebral cortical and cerebellar microglias. Note that the concentrations of IL-1β, IL-6, TNF-α, and MCP-1 were raised in the media of cerebral cortical microglias (**a1**, **b1**) and cerebellar microglias (**c1**, **d1**) treated with sera or IgG in the OMS + NB group; consistently, the expression of nitric oxide (NO) was also enhanced in the media of cerebral cortical microglias (**a2**, **b2**) and cerebellar microglias (**c2**, **d2**) treated with sera or IgG in the OMS + NB group, whereas incubation with sera or IgG from children with only NB had no such effect (**a**–**d**). ^**^p < 0.01, ^***^p < 0.001, one-way ANOVA, *n* = 10 (health control), *n* = 20 (NB), *n* = 10 (OMS + NB)

To further investigate the effects of serum IgG from children with OMS and NB on microglial activation, the concentrations of proinflammatory cytokines (including IL-1β, IL-6, TNF-α, and MCP-1) and NO in the media of cerebral cortical and cerebellar microglias were detected by ELISA. The releases of IL-1β, IL-6, TNF-α, and MCP-1 from cerebral cortical microglias exposed to OMS + NB sera were elevated (IL-1β: 105.4 ± 10.48 pg/ml OMS + NB, 43.99 ± 4.29 pg/ml NB, 39.83 ± 5.64 pg/ml healthy control, *p* < 0.001 vs NB, *p* < 0.001 vs healthy control; IL-6: 107.8 ± 11.20 pg/ml OMS + NB, 61.61 ± 3.67 pg/ml NB, 49.11 ± 5.53 pg/ml healthy control, *p* < 0.01 vs NB, *p* < 0.001 vs healthy control; TNF-α: 169.1 ± 14.32 pg/ml OMS + NB, 100.3 ± 9.15 pg/ml NB, 90.24 ± 6.76 pg/ml healthy control, *p* < 0.001 vs NB, *p* < 0.001 vs healthy control; MCP-1: 294.1 ± 9.30 pg/ml OMS + NB, 181.7 ± 7.93 pg/ml NB, 184.7 ± 9.59 pg/ml healthy control, *p* < 0.001 vs NB, *p* < 0.001 vs healthy control), whereas the incubation with NB sera had no such effect (Fig. 2a1). Furthermore, the levels of IL-1β, IL-6, TNF-α, and MCP-1 secreted from cerebral cortical microglias were increased to 109.6 ± 12.09 pg/ml, 133.2 ± 11.28 pg/ml, 225.3 ± 9.84 pg/ml, and 324.2 ± 6.68 pg/ml after incubation with OMS + NB IgG (*p* < 0.001 vs NB, *p* < 0.001 vs healthy control, Fig. 2b1). In cerebellar microglias, similar impacts of sera and IgG were observed (Fig. 2c1, d1). Consistent with the results of cytokines, NO expression was also improved in the media of cerebral cortical microglias (Fig. 2a2, b2) and cerebellar microglias (Fig. 2c2, d2) treated with OMS + NB sera or IgG. Together with the expression of CD11b, these results suggested that the activation of cultured cerebral cortical and cerebellar microglias was upregulated by serum IgG from children with OMS and NB.

**Fig. 3:**
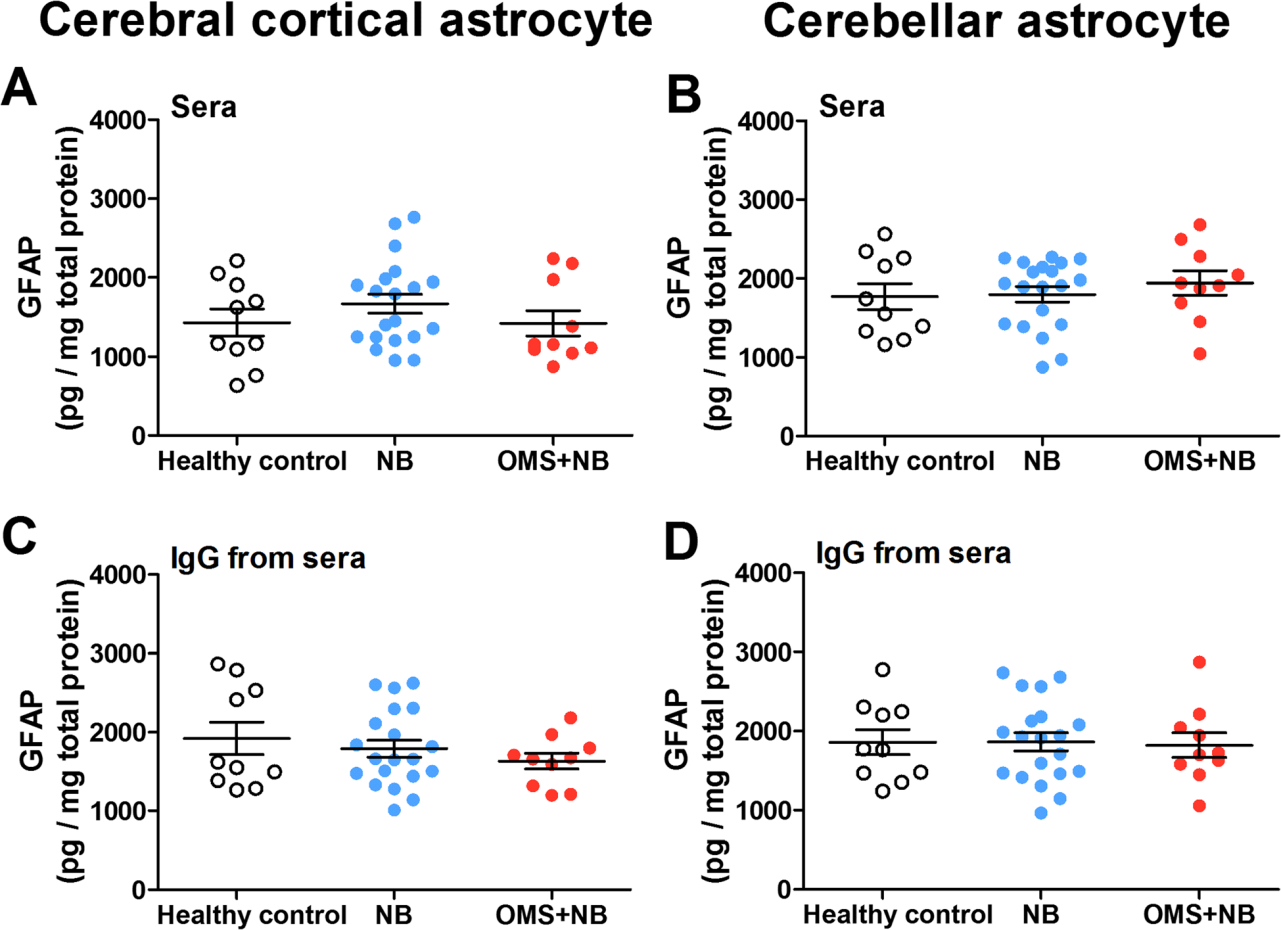
GFAP concentration in cerebral cortical and cerebellar astrocytes incubated with OMS + NB sera or IgG. Note that the expression of GFAP, a marker of astrocytic activation, was not significantly changed in cerebral cortical and cerebellar astrocytes incubated with sera (**a**, **b**) or IgG (**c**, **d**) from children with OMS and NB compared with those from NB patients and healthy control at least under our experimental design. *p* > 0.05, one-way ANOVA, *n* = 10 (health control), *n* = 20 (NB), and *n* = 10 (OMS + NB)

In order to explore whether serum IgG-enhanced activation is specific to microglias or common to glial cells in the CNS, we detected the activation of cerebral cortical and cerebellar astrocytes. Using the marker of astrocytic activation, we found that the expression of GFAP was not significantly changed in cerebral cortical astrocytes (Fig. 3a, c) and cerebellar astrocytes (Fig. 3b, d) incubated with sera or IgG from the OMS + NB group compared with those from the NB group and healthy control group at least under our experimental conditions, suggesting that the enhancement of activation induced by IgG from children with OMS and NB is specific to microglias, but not astrocytes.

**Fig. 4:**
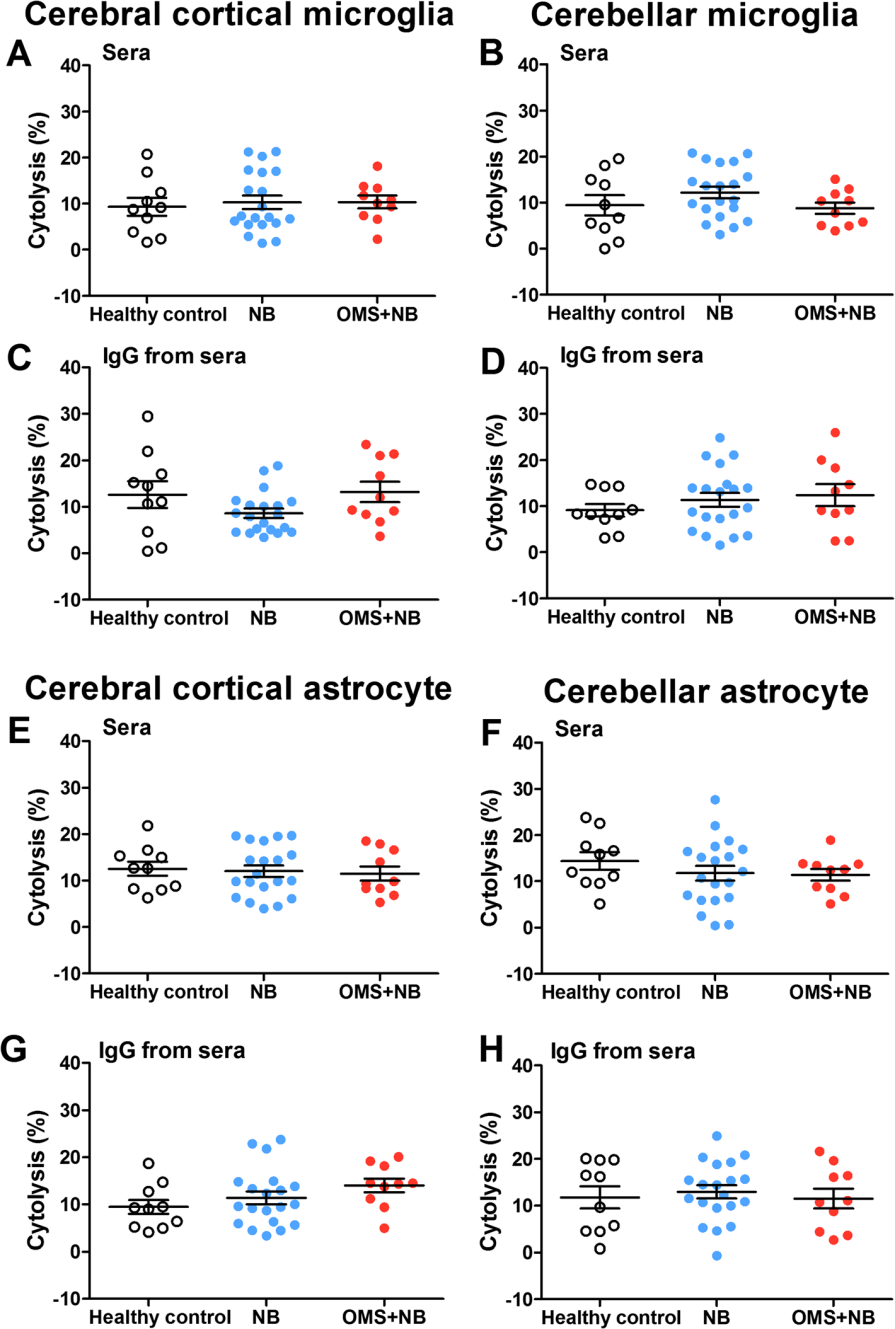
The cytolysis of cerebral cortical and cerebellar glial cells incubated with OMS + NB sera or IgG. Note that neither the cytolysis of cerebral cortical and cerebellar microglias treated with sera (**a**, **b**) or IgG (**c**, **d**) from patients with pediatric OMS and NB, nor the cytolysis of cerebral cortical and cerebellar astrocytes treated with sera (**e**, **f**) or IgG (**g**, **h**) from patients with pediatric OMS and NB was statistically influenced at least under our experimental design. *p* > 0.05, one-way ANOVA, *n* = 10 (health control), *n* = 20 (NB), and *n* = 10 (OMS + NB)

Previously, we have revealed that serum IgG from children with OMS and NB induces cytolysis in cultured neurons [6], whether serum IgG impacts the cytolysis of microglias or astrocytes needs further study. We observed that neither the cytolysis of cerebral cortical microglias (Fig. 4a, c) and cerebellar microglias (Fig. 4b, d) treated with OMS + NB sera or IgG, nor the cytolysis of cerebral cortical astrocytes (Fig. 4e, g) and cerebellar astrocytes (Fig. 4f, h) treated with OMS + NB sera or IgG was statistically changed, which suggested that preincubation with sera or IgG from children with OMS and NB specially upregulates microglial activation rather than the cytolysis of microglias and astrocytes at least under our experimental conditions.

### The cytolysis of neurons is induced by conditioned media from microglias treated with OMS + NB IgG

**Fig. 5:**
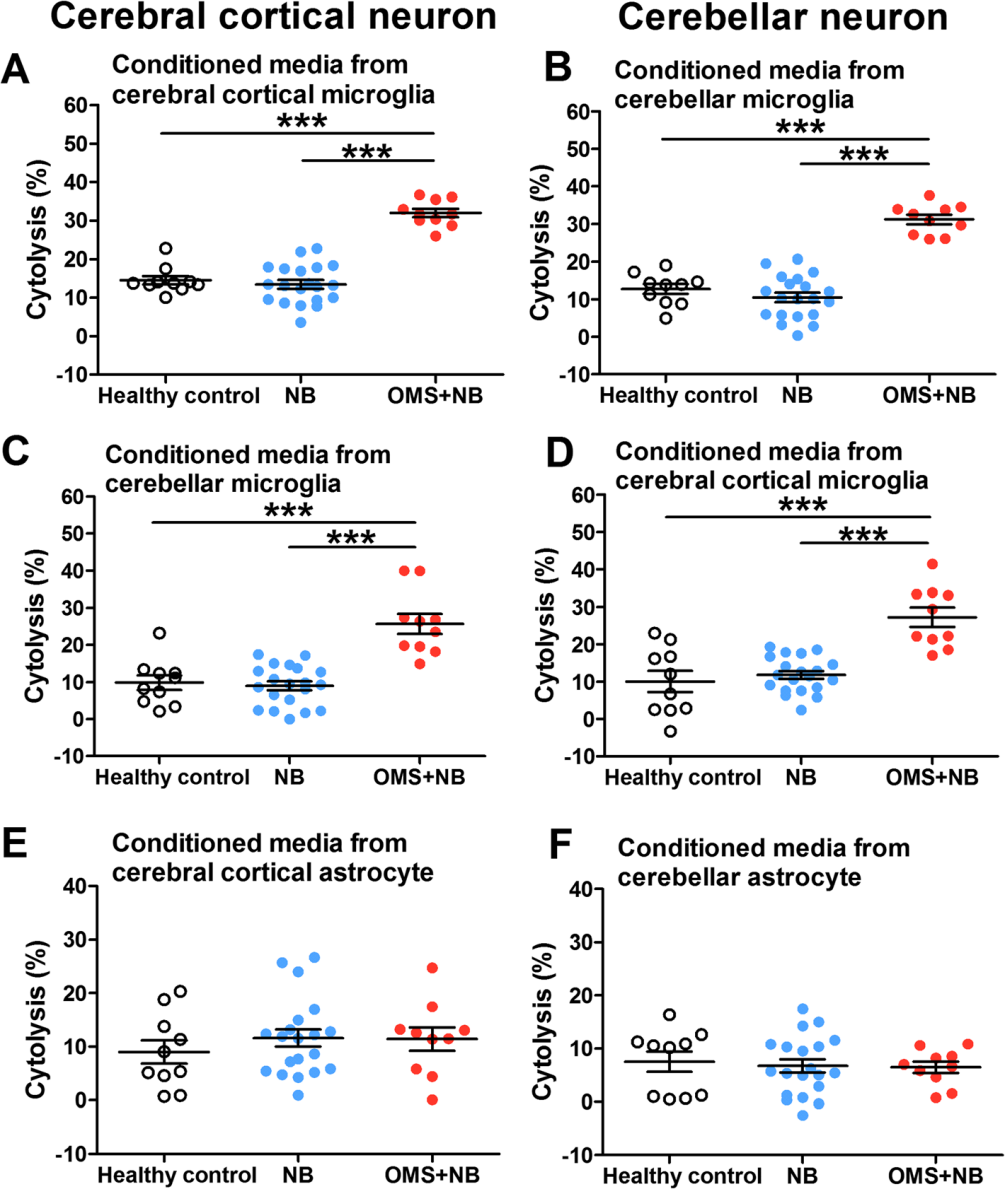
Neuronal cytolysis induced by conditioned media from cerebral cortical and cerebellar microglias. After microglias incubated with OMS + NB IgG, conditioned media were collected, got rid of the remaining IgG, and replaced the culture media of neurons. Note that preincubation with conditioned media from cerebral cortical and cerebellar microglias enhanced the cytolysis of neurons in the same brain regions (**a**, **b**) or exchanged brain regions (**c**, **d**), while no alteration was observed in the cytolysis of neurons incubated with conditional media from astrocytes (**e**, **f**). ^***^p < 0.001, one-way ANOVA, *n* = 10 (health control), *n* = 20 (NB), and *n* = 10 (OMS + NB)

It has been established that activated microglias contribute to neuron death by secreting various neurotoxic molecules in multiple disorders [8, 17] and serum IgG from patients with OMS and NB enhances neuronal death [6, 7, 36], we therefore explored whether neuronal cytolysis is enhanced by microglial activation in OMS. After getting rid of the remaining IgG, serum IgG-treated microglia conditioned media were collected and were replaced the culture media of neurons. As expected, incubation with conditioned media from cerebral cortical and cerebellar microglias increased the cytolysis of neurons in the same brain regions (cerebral cortical neuron: 31.95 ± 1.09% OMS + NB, 13.45 ± 1.12% NB, 14.53 ± 1.08% healthy control; cerebellar neuron: 31.21 ± 1.25% OMS + NB, 10.50 ± 1.27% NB, 12.75 ± 1.34% healthy control; *p* < 0.001 vs NB, *p* < 0.001 vs healthy control, Fig. 5a, b). Moreover, conditioned media from cerebral cortical microglias to cerebellar neurons or the exchanged situation had similar results (cerebral cortical neuron: 25.67 ± 2.71% OMS + NB, 9.00 ± 1.21% NB, 9.85 ± 1.95% healthy control; cerebellar neuron: 27.24 ± 2.55% OMS + NB, 11.79 ± 1.06% NB, 10.03 ± 2.86% healthy control; *p* < 0.001 vs NB, *p* < 0.001 vs healthy control, Fig. 5c, d). Conversely, we observed no alteration in the cytolysis of neurons treated with conditional media from astrocytes (Fig. 5e, f). Taken together, these results suggested that conditioned media from cerebral cortical or cerebellar microglias rather than astrocytes induce the cytolysis of neurons.

### NO/sGC/PKG signaling contributes to conditioned media-induced neuronal cytolysis

**Fig. 6:**
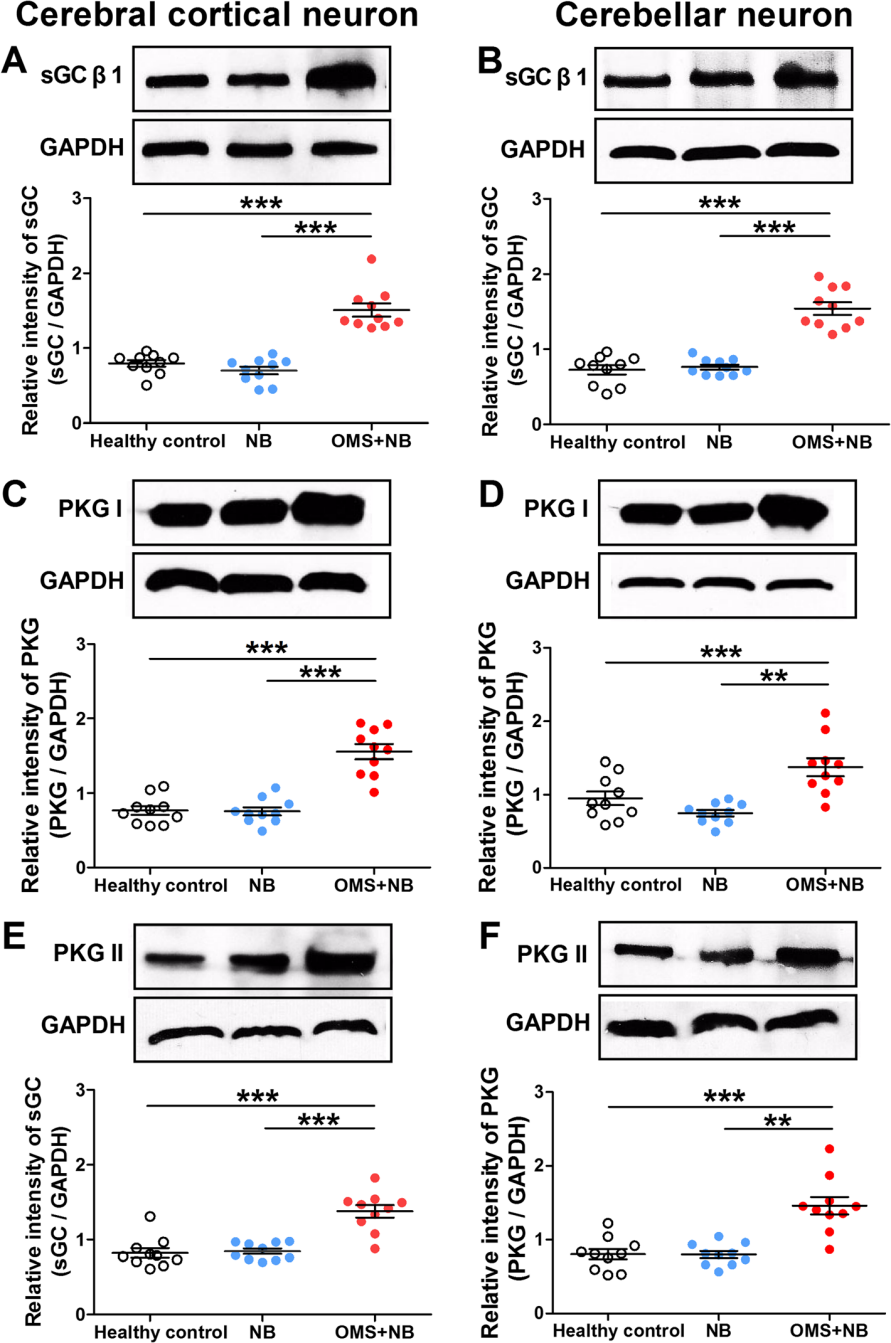
The expression of sGC and PKG in cultured neurons incubated with conditioned media. Note that the protein expression of β1 subunit of sGC (**a**, **b**), PKG I (**c**, **d**), and PKG II (**e**, **f**) was increased in cerebral cortical and cerebellar neurons incubated with conditioned media from microglias treated with OMS + NB IgG. ^**^p < 0.01, ^***^p < 0.001, one-way ANOVA, *n* = 10/group

To investigate the mechanisms of cytolysis induced by conditioned media from microglias treated with OMS + NB IgG, we focus on the NO/sGC/PKG pathway, for its role in neuronal death is well documented [21–23] and the production of NO from microglias was raised in the OMS + NB group (Fig. 2a2–d2). We first examined the alterations of sGC and PKG abundance. Enzymatic activity of sGC needs its β subunit, and although two types of β subunit (β1 and β2) have been cloned, only β1 subunit has been shown to exist at the protein level in the brain [37]. Two types of PKG (PKG I and PKG II) have been identified in mammalian tissues, both are related to cell apoptosis, proliferation [38, 39], and brain function [40, 41]. Our results showed that the protein expression of β1 subunit of sGC was upregulated in cerebral cortical and cerebellar neurons after incubation with conditioned media of the OMS + NB group (cerebral cortical neuron: 1.54 ± 0.08 OMS + NB, 0.76 ± 0.03 NB, 0.72 ± 0.06 healthy control, *p* < 0.001 vs NB, *p* < 0.001 vs healthy control; cerebellar neuron: 1.56 ± 0.10 OMS + NB, 0.76 ± 0.05 pg/ml NB, 0.77 ± 0.06 pg/ml healthy control, *p* < 0.001 vs NB, *p* < 0.001 vs healthy control; Fig. 6a, b), and the expression of PKG I and PKG II was also increased (Fig. 6c–f).

**Fig. 7:**
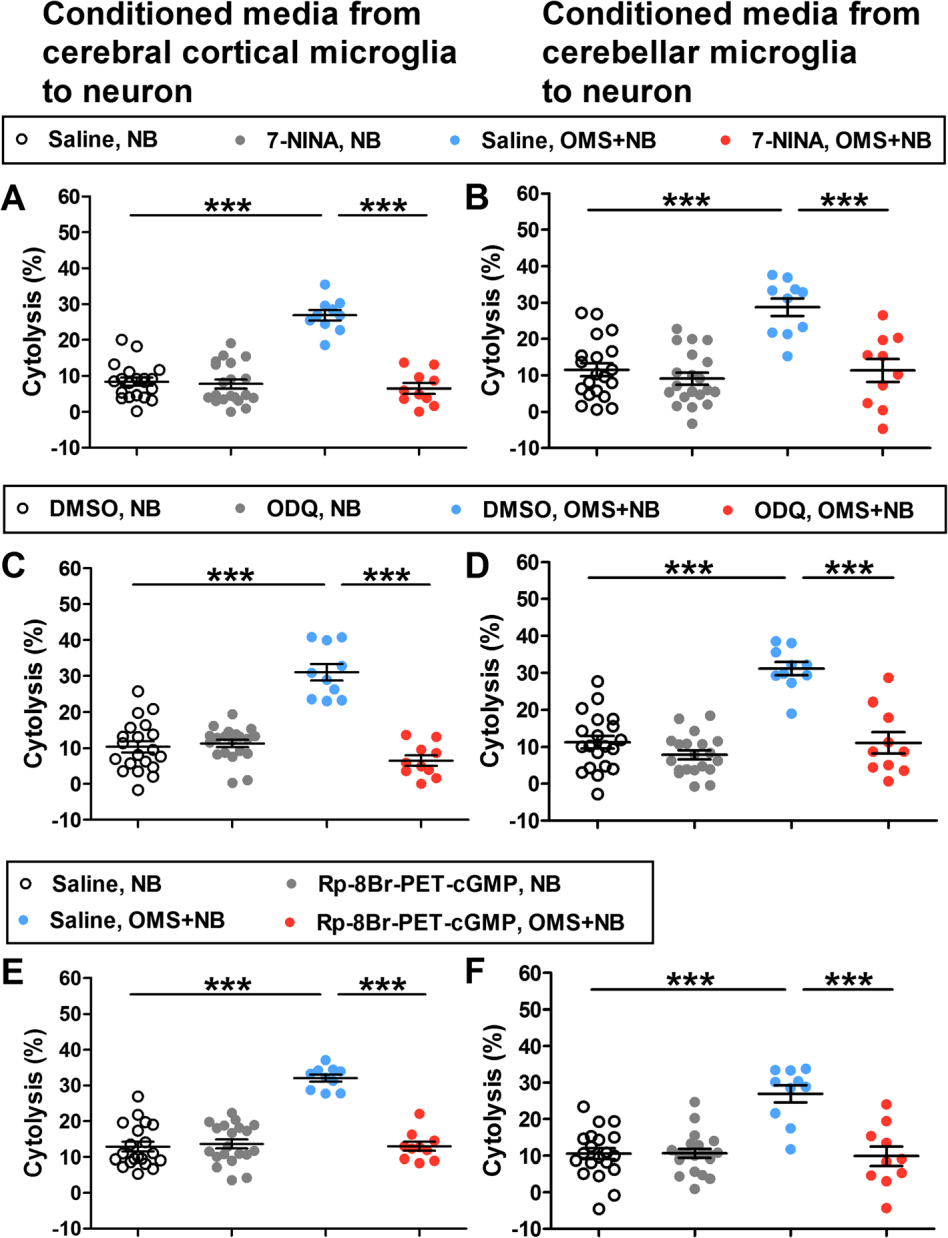
The alleviation of neuronal cytolysis by pretreatment with pharmacological inhibitors of the NO cascade. Note that the cytolysis of cerebral cortical neurons (**a**) and cerebellar neurons (**b**) induced by conditioned media from microglias treated with OMS + NB IgG was relieved by the NO synthesis inhibitor 7-NINA, the cytolysis of cerebral cortical neurons (**c**) and cerebellar neurons (**d**) was reduced by the sGC inhibitor ODQ, and the cytolysis of cerebral cortical neurons (**e**) and cerebellar neurons (**f**) was abrogated by Rp-8Br-PET-cGMP, an inhibitor of PKG. ^***^p < 0.001, one-way ANOVA, *n* = 20 (vehicle, NB; inhibitor, NB), *n* = 10 (vehicle, OMS + NB; inhibitor, OMS + NB)

Next, the effects of inhibitors of NO synthesis and NO-activated intracellular pathway were assessed. Pretreatment with the NO synthesis inhibitor 7-NINA to microglias before OMS + NB IgG alleviated the cytolysis of cerebral cortical and cerebellar neurons (cerebral cortical neuron: 6.50 ± 1.46% 7-NINA, OMS + NB vs 26.91 ± 1.45% saline, OMS + NB, *p <* 0.001; 26.91 ± 1.45% saline, OMS + NB vs 8.36 ± 1.10% saline, NB, *p <* 0.001; cerebellar neuron: 11.30 ± 3.14% 7-NINA, OMS + NB vs 28.72 ± 2.43% saline, OMS + NB, *p <* 0.001; 28.72 ± 2.43% saline, OMS + NB vs 11.50 ± 1.83% saline, NB, *p <* 0.001; Fig. 7a, b). Similarly, pretreatment with the sGC inhibitor ODQ or the PKG inhibitor Rp-8Br-PET-cGMP ameliorated the cytolysis of cerebral cortical and cerebellar neurons (Fig. 7c–f).

**Fig. 8:**
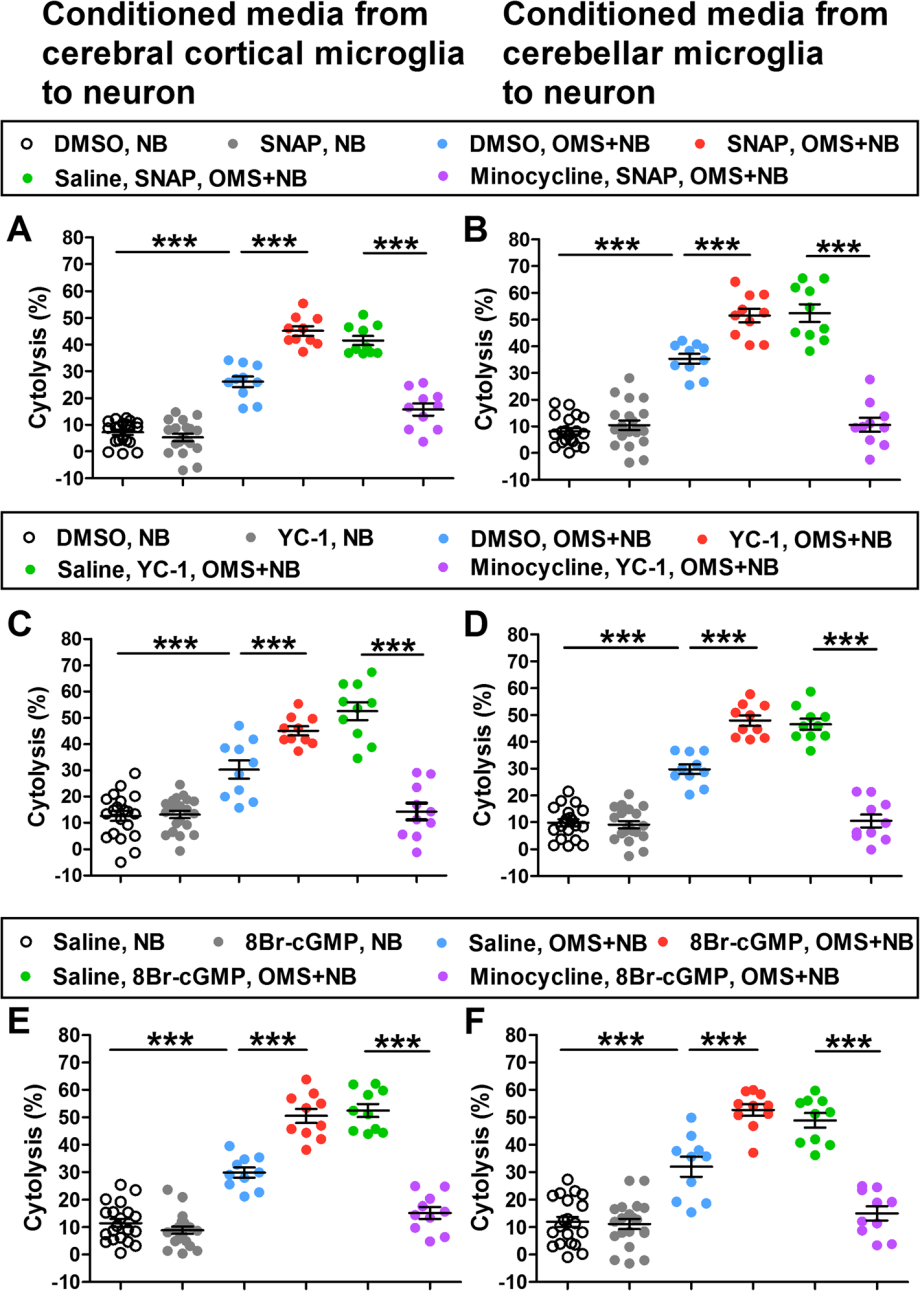
The exaggeration of neuronal cytolysis by pretreatment with pharmacological activators of the NO pathway. Note that the cytolysis of cerebral cortical neurons (**a**) and cerebellar neurons (**b**) induced by conditioned media from microglias treated with OMS + NB IgG was exacerbated by the NO-donor SNAP, and the cytolysis of cerebral cortical neurons (**c**) and cerebellar neurons (**d**) was also upregulated by YC-1, an activator of sGC. Similarly, the PKG activator 8Br-cGMP had exaggerated effects on the cytolysis of cerebral cortical neurons (**e**) and cerebellar neurons (**f**). The exaggerated effects of SNAP, YC-1, or 8Br-cGMP and neuronal cytolysis induced by conditioned media were suppressed by pretreatment with minocycline, an inhibitor of microglias (**a**–**f**). ^***^p < 0.001, one-way ANOVA, *n* = 20 (vehicle, NB; activator, NB), *n* = 10 (vehicle, OMS + NB; activator, OMS + NB; saline, activator, OMS + NB; minocycline, activator, OMS + NB)

Furthermore, the effects of activators of NO synthesis and NO-activated intracellular pathway were examined. The cytolysis of cerebral cortical neurons was exaggerated after incubation with conditioned media from microglias pretreated with the NO-donor SNAP 30 min before OMS + NB IgG (45.05 ± 1.74% SNAP, OMS + NB vs 26.09 ± 2.08% DMSO, OMS + NB, *p <* 0.001; 26.09 ± 2.08% DMSO, OMS + NB vs 7.12 ± 0.98% DMSO, NB, *p <* 0.001; Fig. 8a), and the cytolysis of cerebellar neurons was exacerbated by treatment with SNAP before OMS + NB IgG (51.51 ± 2.55% SNAP, OMS + NB vs 35.27 ± 1.87% DMSO, OMS + NB, *p <* 0.001; 35.27 ± 1.87% DMSO, OMS + NB vs 8.06 ± 1.21% DMSO, NB, *p <* 0.001; Fig. 8b). Pretreatment with the activator of sGC or PKG, namely YC-1 or 8Br-cGMP, also exacerbated cerebral cortical and cerebellar neuronal cytolysis induced by conditioned media from microglias stimulated with OMS + NB IgG (Fig. 8c–f). In addition, pretreatment with minocycline, an inhibitor of microglia, almost completely blocked the exacerbatory effects of these abovementioned activators and neuronal cytolysis induced by IgG-treated microglia conditioned media (Fig. 8a–f). Collectively, these results suggested that the NO/sGC/PKG cascade plays a vital role in neuronal cytolysis induced by conditioned media from microglias treated with serum IgG from children with OMS and NB, which depends on the activation of microglias.

**Fig. 9:**
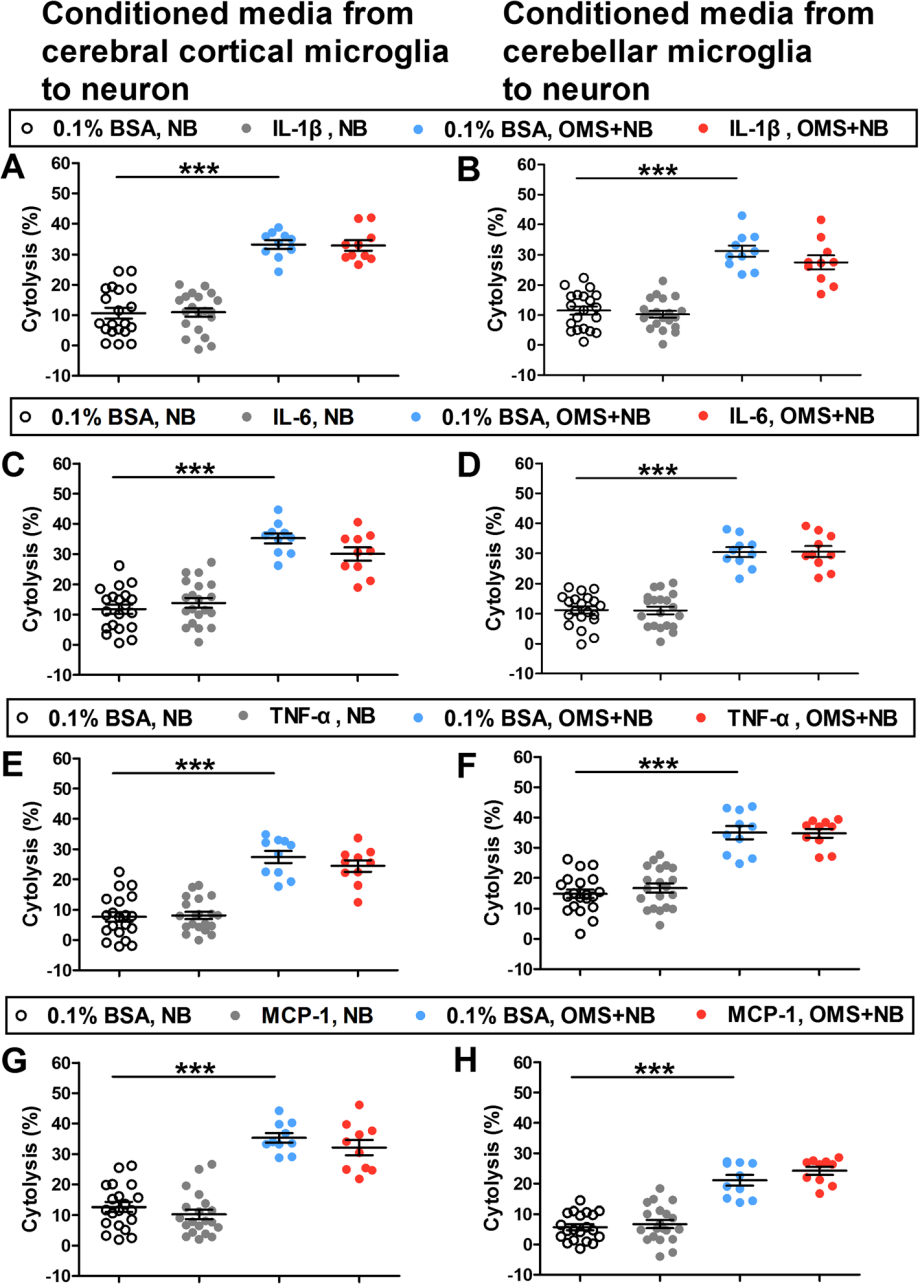
The effects of cytokines on neuronal cytolysis induced by conditioned media from microglias. Note that the cytolysis of cerebral cortical neurons (**a**) and cerebellar neurons (**b**) induced by conditioned media from microglias preincubated with OMS + NB IgG was not influenced by IL-1β, and the cytolysis of cerebral cortical neurons (**c**) and cerebellar neurons (**d**) was also not changed by IL-6. Consistently, the cytolysis of cerebral cortical neurons (**e**) and cerebellar neurons (**f**) was not affected by TNF-α, and the cytolysis of cerebral cortical neurons (**g**) and cerebellar neurons (**h**) was not changed by MCP-1. *p* > 0.05; 0.1% BSA, OMS + NB vs cytokine, OMS + NB. ^***^p < 0.001; 0.1% BSA, NB vs cytokine, NB. One-way ANOVA, *n* = 20 (0.1% BSA, NB; cytokine, NB), *n* = 10 (0.1% BSA, OMS + NB; cytokine, OMS + NB)

The expression of proinflammatory cytokines in the culture medium of microglias incubated with OMS + NB IgG (Fig. 2a1–d1) and in CSF of OMS children [10] were upregulated, which raises the possibility that cytokines may have similar effects on neuronal cytolysis. Unexpectedly, our results showed that the cytolysis of cerebral cortical and cerebellar neurons induced by conditioned media from microglias preincubated with OMS + NB IgG was not influenced by IL-1β (cerebral cortical neuron: 32.89 ± 1.70% IL-1β, OMS + NB vs 33.23 ± 1.37% 0.1% BSA, OMS + NB, *p >* 0.05; 33.23 ± 1.37% 0.1% BSA, OMS + NB vs 10.65 ± 1.76% 0.1% BSA, NB, *p <* 0.001; cerebellar neuron: 27.55 ± 2.32% IL-1β, OMS + NB vs 31.26 ± 1.88% 0.1% BSA, OMS + NB, *p >* 0.05; 31.26 ± 1.88% 0.1% BSA, OMS + NB vs 11.48 ± 1.39% 0.1% BSA, NB, *p <* 0.001; Fig. 9a, b). Also, enhanced cytolysis of cerebral cortical and cerebellar neurons was not affected by IL-6, TNF-α, or MCP-1 (Fig. 9c–h). These results suggested that the application of cytokines does not have similar effects of NO on the cytolysis of neurons induced by IgG-treated microglia conditioned media.

### The activation of cerebral cortical and cerebellar microglias may depend on the Fc fragment of serum IgG rather than the Fab fragment

**Fig. 10:**
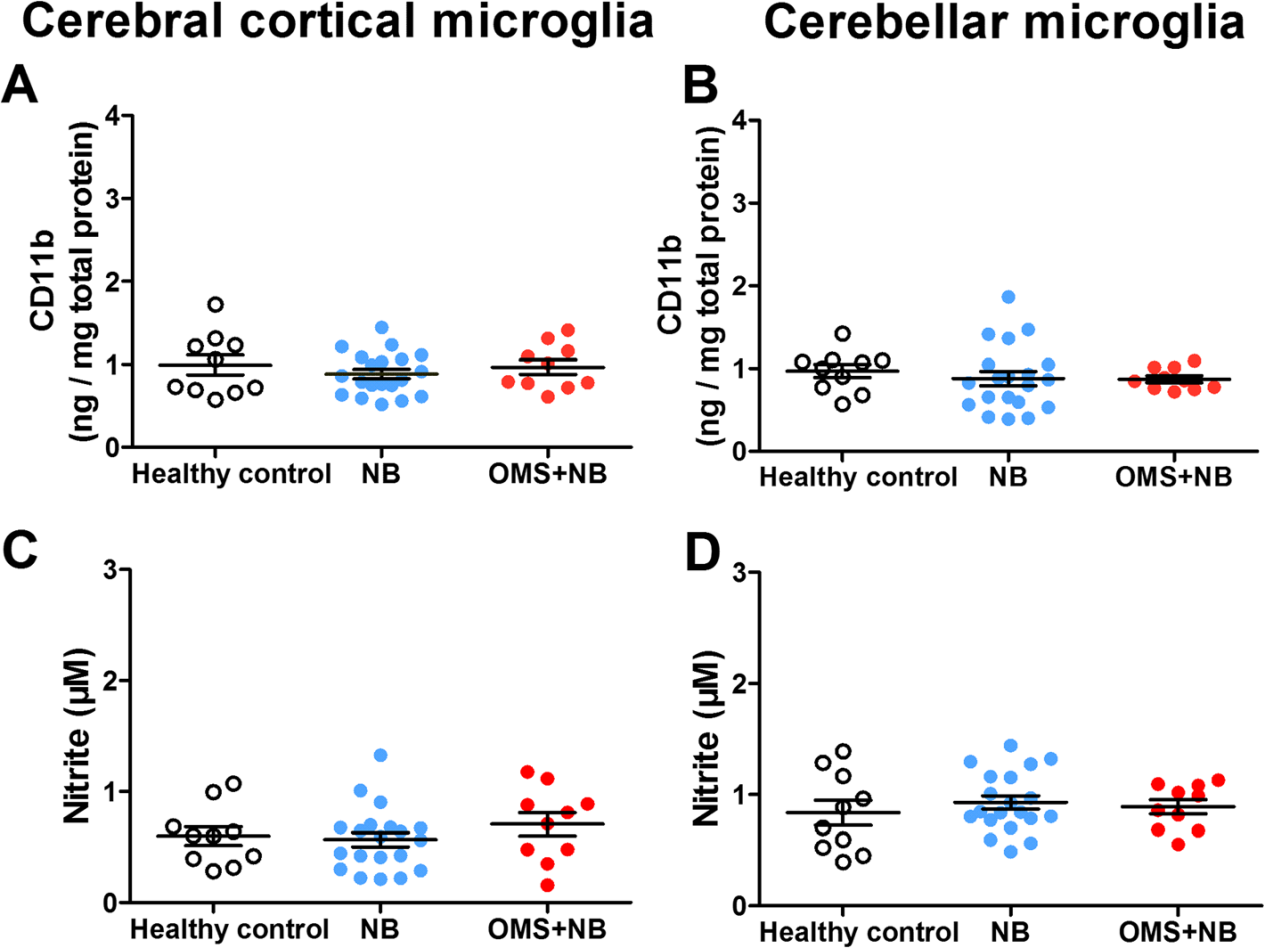
The Fab fragment of serum IgG cannot induce microglial activation. Note that the expression of CD11b and NO was not significantly changed in cerebral cortical microglias (**a**, **b**) and cerebellar microglias (**c**, **d**) incubated with the Fab fragment of IgG from children with OMS and NB compared with that from NB patients and healthy control. *p* > 0.05, one-way ANOVA, *n* = 10 (health control), *n* = 20 (NB), and *n* = 10 (OMS + NB)

IgG contains the Fab fragment and the Fc fragment, and the Fab fragment combines with targeted antigens, while the Fc fragment interacts with Fcγ receptor (FcγR). FcγR is expressed on the surface of microglias and other immune effector cells and mediates immune reactions in the brain. In order to explore whether increased microglial activation depends on the Fab fragment or the Fc fragment of serum IgG, we detected the effects of the Fab fragment on the expression of CD11b in microglias and the concentration of NO in the culture medium of microglias. The results showed that the expression of CD11b was not significantly changed in cerebral cortical and cerebellar microglias incubated with the Fab fragment from the OMS + NB group compared with that from the NB group and healthy control group (Fig. 10a, b). Consistently, the release of NO was not significantly changed in cerebral cortical and cerebellar microglias (Fig. 10c, d). These results suggesting that microglial activation induced by IgG from children with OMS and NB may depend on the Fc fragment of IgG, but not the Fab fragment.

## Discussion

### The IgG fraction of sera from children with OMS and NB gives rise to the activation of cultured cerebral cortical and cerebellar microglias

Neuroinflammation in the CNS, a dominant physiological function of microglias and astrocytes, is a common clinical feature in children with OMS [9]. Moreover, the expression of microglial marker soluble CD14 and proinflammatory cytokines is enhanced in CSF from patients with pediatric OMS [10, 11]. Additionally, serum IgG from patients or autoantibody existed in patients directly enhances microglial activation in PD [12], ALS [13], and SLE [14–16] or initially binds with astrocytes or neurons and further indirectly affects microglial activation [42, 43]. Autoantibodies are also detectable in sera and CSF of children with OMS [25, 26] and may be contained in serum IgG from children with OMS and NB in our study, although we did not identify these autoantibodies and autoantibodies include IgM besides IgG. Thus, we hypothesized that serum IgG from children with OMS and NB may impact the activation of microglias. As expected, our results revealed that sera or IgG from children with OMS and NB upregulated the activation of cultured cerebral cortical and cerebellar microglias.

Conversely, commercially available human IgG, or IgG from children with JIA or anti-NMDAR encephalitis, had no such effect, suggesting that the upregulation of microglial activation induced by serum IgG from children with OMS and NB is not simply induced by increased dose of IgG, is not common to all IgG-related diseases, and is not common to all autoantibody-mediated disorders of the CNS, indicating specific changes in pediatric OMS at least to some degree. Consistently, we and others found that IgG-induced neuronal cytolysis occurs in pediatric OMS rather than adult OMS [6, 7]. Notably, increased microglial activation is not specific to only OMS, since previous literatures have documented that serum IgG from patients with PD [12] or ALS [13] enhances the activation of microglia and the production of NO, and serum IgG from patients with SLE induced behavioral changes is mediated by microglial activation [14–16]. Moreover, several autoantibodies found in patients with OMS [44–47] also exist in other diseases, such as autoantibody against glycine receptor in progressive encephalomyelitis with rigidity and myoclonus [46, 48] or autoantibody against glutamic acid decarboxylase in stiff-person syndrome [48].

Unlike microglial activation, astrocytic activation by serum IgG from children with OMS and NB was not observed by us, indicating that microglia reactivity may be a special mechanism. This notion is supported by studies demonstrating that human immunodeficiency virus infection increases microglial activation but not astrocytic activation [14], and administration of AMD3100 alleviates the pathology of ALS by decreasing microglial activation without affecting astrocytes [17]. However, we cannot completely exclude the role of astrocytes, and the activation of astrocytes may be improved by conditioned media from microglia treated with serum IgG from OMS patients rather than direct stimulation by serum IgG. Furthermore, distinct from the upregulated cytolysis of neurons [6], we observed that the cytolysis of microglias and astrocytes by serum IgG from children with OMS and NB was not changed.

Some cases of pediatric OMS are paraneoplastic and are associated with NB, although varied percentages have been reported [1–5]. The remaining cases are believed to be post-infectious or resulted from NB that has regressed prior to onset of symptoms [48]. Adults with non-paraneoplastic OMS have better outcomes with fewer relapses [47], while children with paraneoplastic and non-paraneoplastic OMS have no significant difference in viral-like prodrome and neurological outcome [1, 49]. However, whether there is a distinction of paraneoplastic and non-paraneoplastic childhood OMS is still unclear. Our present and previous studies showed that microglial activation or neuronal cytolysis can be induced by serum IgG from children with OMS and NB, but not children with only NB [6], indicating that the IgG fraction may be involved in the pathogenesis of OMS rather than NB. However, we did not obtain sera from children with OMS and without NB; therefore, we cannot completely exclude the possibility that the cytotoxicity induced by serum IgG from children with OMS and NB may be synergistic effects of OMS and NB.

### The NO/sGC/PKG pathway takes part in neuronal cytolysis induced by conditioned media from microglias treated with IgG from children with OMS and NB

A growing body of evidence implicates that microglias in the active state can release various neurotoxic molecules [8, 17], causing the loss of neurons in neurodegenerative diseases, including Alzheimer’s disease [18], ALS [20], and retinal degeneration [19]. Moreover, we and others have previously demonstrated that sera or IgG from patients with pediatric OMS and NB induces the cytolysis of cerebellar granular and cerebral cortical neurons [6, 7]. Consistently, here our results showed that neuronal cytolysis was elevated by conditional media from microglias treated with IgG from children with OMS and NB. Notably, conditioned media from microglias were applied to cultured neurons instead of co-culture of microglias and neurons, and conditioned media were used to cultured neurons after IgG were filtered out, to avoid the direct effects of serum IgG on neurons [6].

NO, synthesized and released by activated microglias, can activate sGC and PKG in neurons, leading to neuronal death by mitochondrial dysfunction [21, 22] and increased neuronal susceptibility to mitochondrial dysfunction [23], which is similar to its function in pancreatic tissues [49]. On the other hand, exposure to NO has anti-apoptotic function through sGC and PKG in both neuronal and myocardial tissues [50, 51], and the opposite influences of NO intracellular signal may be caused by the way (transient or sustained) and concentration of NO, as well as cell type. In the present study, our results showed the NO/sGC/PKG cascade was a positive regulator of neuronal cytolysis induced by conditioned media from microglias treated with IgG from children with OMS and NB.

**Fig. 11:**
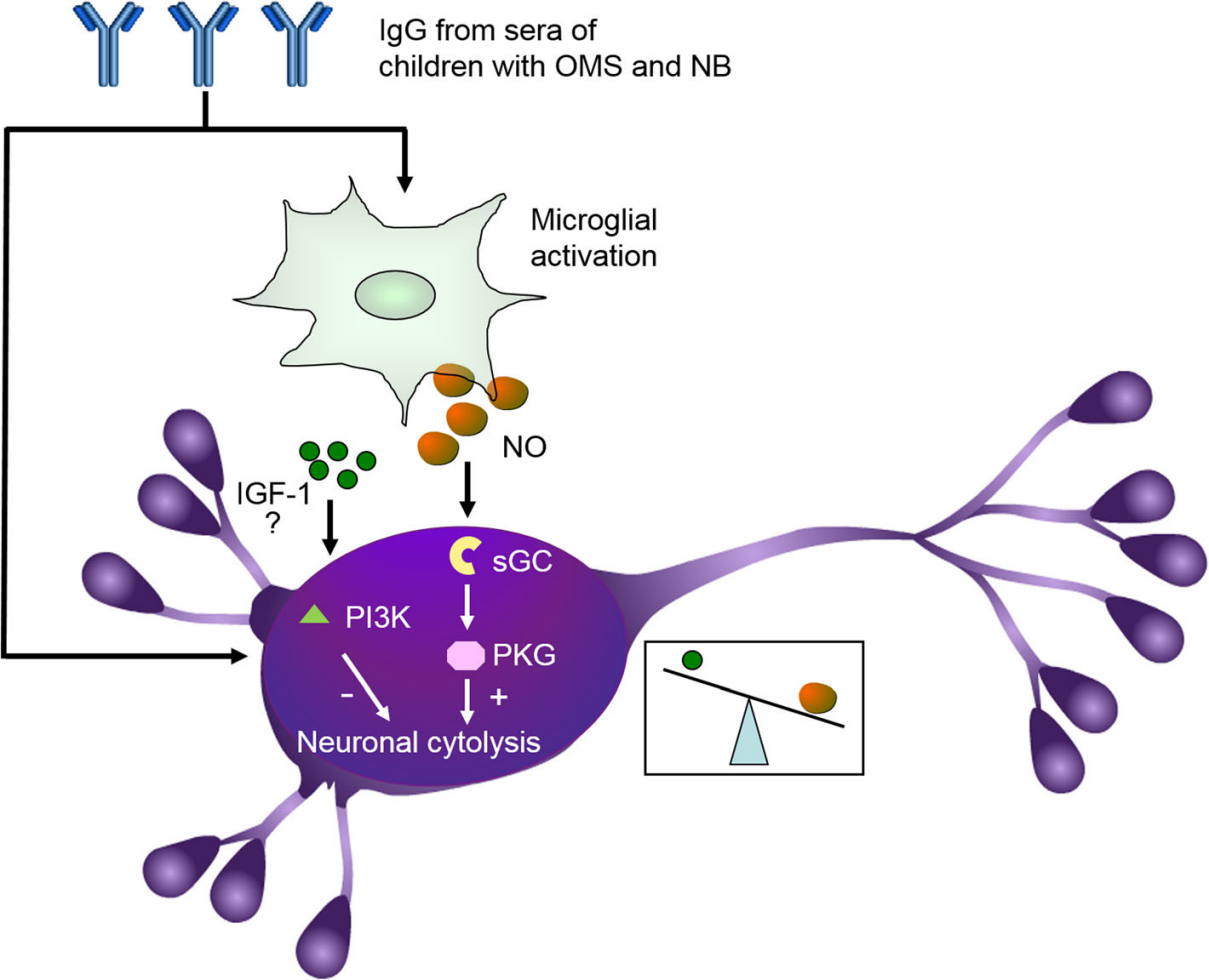
Schematic diagram illustrating the possible mechanisms of serum IgG-induced cytolysis of neurons. As we previously demonstrated, serum IgG from children with OMS and NB directly enhances the cytolysis of cultured cerebral cortical and cerebellar neurons. On the other hand, serum IgG also increases the activation of cultured microglias, leading to the upregulation of NO, which subsequently activate sGC and PKG in neurons, thereby inducing neuronal cytolysis. Although at the same time IGF-1/PI3K signaling may be activated to alleviate neuronal lysis, the impact of the NO/sGC/PKG pathway may be more predominant

Previously, we have documented that IGF-1/PI3K signaling is activated as a compensative mechanism to alleviate neuronal cytolysis directly induced by IgG from sera of children with OMS and NB [6]. Here, we found that the concentration of PI3K was elevated in cerebral cortical and cerebellar neurons incubated with conditioned media from microglias treated with IgG from children with OMS and NB. We also found that exogenous IGF-1 alleviated the cytolysis of neurons incubated with conditioned media, which was attenuated by the PI3K inhibitor (see Additional file 1: Figure S1). These results indicated that IGF-1/PI3K signaling may be compensatively activated to alleviate neuronal lysis induced by IgG through microglias, which is similar to the direct effects of IgG on neuronal lysis. Taken together, IgG from children with OMS and NB increases the activation of microglias, leading to the upregulation of NO, which subsequently activates sGC and PKG in neurons to induce neuronal lysis, at the same time IGF-1/PI3K signaling may be compensatively activated to alleviate neuronal lysis; however, the role of NO and its intracellular cascade seems to be more predominant (Fig. 11).

Interestingly, our results showed that neuronal cytolysis induced by conditioned media from microglias via NO was almost completely attenuated by the inhibitor of microglia, minocycline. Minocycline has been clinically used for treating infections as an antibiotic, providing a systemic anti-inflammatory effect. Moreover, it produces the anti-inflammatory response in the CNS via microglias and has neuroprotective properties in neurodegenerative diseases, mental illnesses, and others [52–56]. In particular, minocycline delays motor alterations, inflammation, and apoptosis in experimental models of PD and ALS [56]. Although there are controversies about its efficacy, the relative safety and tolerability of minocycline have led to the launching of various clinical trials; for instance, it may be a possible treatment for patients with acute ischemic stroke [52, 53]. Thus, the administration of minocycline might be an efficient way to treat pediatric OMS.

Although we found that proinflammatory cytokines, namely IL-1β, IL-6, TNF-α, and MCP-1, were elevated in the media of microglias treated with IgG from patients with pediatric OMS and NB, and others reported that IL-6 in CSF of untreated OMS patients and IL-1 receptor antagonist in CSF of intravenous immune globulin-treated OMS patients are upregulated [10], the cytolysis of cerebral cortical and cerebellar neurons induced by conditioned media from microglias preincubated with serum IgG was not influenced by these proinflammatory cytokines, indicating a specific effect of the NO pathway on neuronal cytolysis induced by microglial activation. Possible explanations are that cytokines secreted from microglias may take part in other functions rather than neuronal cytolysis, such as the recruitment of immune cells [8], and cytokines enhanced in patients with OMS may be produced by other cells, not microglias.

### Microglial activation induced by serum IgG from children with OMS and NB may be through the Fc fragment of IgG

FcγR, expressed on the surface of microglias, interacts with the Fc fragment of IgG to release proinflammatory cytokines, thereby mediating immune reactions. Several previous studies have documented that IgG combines with FcγR to activate microglias and thus takes part in many diseases. For example, serum IgG from patients with PD significantly induces microglial activation via FcγR in the microglia-supplemented neuronal cultures [12]. Also, in an in vitro PD model, neuron-derived IgG activates microglias through FcγR [57]. Moreover, autoantibodies can activate microglias through FcγR underlie the pathogenesis of autoimmune diseases. For instance, the expression of FcγR crucially contributes to autoantibody-induced tissue injury in experimental epidermolysis bullosa acquisita, an organ-specific autoimmune disease [58]. In agreement with these findings, our results showed that microglial activation may be increased through the Fc fragment of IgG from children with OMS and NB rather than the Fab fragment of IgG. Of interest to note is that we found the activation of microglias was not affected by treatment with commercially available human IgG, IgG from children with JIA or anti-NMDAR encephalitis. These findings are consistent with previous reports that IgG combined with microglial FcγR can secrete different molecules, either proinflammatory or anti-inflammatory, which depends on the origin and content of IgG [12, 57]. Collectively, it is reasonable to speculate that FcγR may be involved in enhanced microglial activation triggered by the Fc fragment of serum IgG from children with OMS and NB.

## Conclusions

In the present study, we demonstrated that incubation with sera or the IgG fraction from children with OMS and NB upregulates the activation of cultured cerebral cortical and cerebellar microglias. Furthermore, neuronal cytolysis is exerted by incubation with conditioned media from microglias treated with IgG from children with OMS and NB. In addition, the NO/sGC/PKG pathway contributes to neuronal cytolysis induced by conditioned media, and neuronal cytolysis can be almost completely suppressed by pretreatment with the microglial inhibitor minocycline, a clinically tested drug. Finally, increased microglial activation may depend on the Fc fragment of serum IgG rather than the Fab fragment. Our data provide solid evidence that serum IgG from children with OMS and NB increases microglial activation, which induces neuronal cytolysis through the NO/sGC/PKG pathway, suggesting that the inhibitor of microglia, such as minocycline, may serve as a plausible therapeutic candidate for pediatric OMS.

## Supplementary information

**Additional file 1: Figure S1.** Effects of IGF-1/PI3K signaling on the cytolysis of neurons induced by conditioned media. The concentration of PI3K was increased in cerebral cortical neurons (a) and cerebellar neurons (b) incubated with conditioned media of the OMS + NB group. The cytolysis of cerebral cortical neurons (c) and cerebellar neurons (d) incubated with conditioned media of the OMS + NB group was alleviated by exogenous IGF-1, which was suppressed by pretreatment with the PI3K inhibitor LY294002. ^***^p < 0.001, one-way ANOVA, n=20 (PBS, NB; IGF-1, NB), n=10 (PBS, OMS+NB; IGF-1 OMS+NB; DMSO, IGF-1, OMS+NB; LY294002, IGF-1, OMS+NB).

## Data Availability

The datasets used and/or analyzed during the current study are available from the corresponding author on reasonable request.

## Abbreviations

7-NINA: 7-Nitroindazole
ALS: Amyotrophic lateral sclerosis
ANOVA: Analysis of variance
BSA: Bovine serum albumin
CD11b: Cluster of differentiation 11b
CNS: Central nervous system
CSF: Cerebrospinal fluid
DMSO: Dimethyl sulfoxide
ELISA: Enzyme-linked immunosorbent assay
GAPDH: Glyceraldehyde 3-phosphate dehydrogenase
GFAP: Glial fibrillary acidic protein
IGF-1: Insulin-like growth factor 1
IL-1β: Interleukin-1β
IL-6: Interleukin-6
JIA: Juvenile idiopathic arthritis
LDH: Lactate dehydrogenase
MCP-1: Monocyte chemoattractant protein-1
NB: Neuroblastoma
NMDA: *N*-methyl-d-aspartate
NO: Nitric oxide
ODQ: 1H-1,2,4 Oxadiazolo-4,3-a quinoxalin-1-one
OMS: Opsoclonus-myoclonus syndrome
PBS: Phosphate buffer saline
PD: Parkinson disease
PI3K: Phosphoinositide 3-kinase
PKG: Protein kinase G
Po-S: Ponceau S solution
sGC: Soluble guanylyl cyclase
SLE: Systemic lupus erythematosus
SNAP: *S*-nitroso-*N*-acetylpenicillamine
TNF-α: Tumor necrosis factor-α
YC-1: 3-5-Hydroxymethyl-2-furyl-1-benzyl-indazole
