# Supplementary material for: Serum IgG-induced microglial activation enhances neuronal cytolysis via the NO/sGC/PKG pathway in children with opsoclonus-myoclonus syndrome and neuroblastoma
Source: J Neuroinflammation. 2020 Jun 16;17:190. doi: 10.1186/s12974-020-01839-9 (PMC7298801; doi:10.1186/s12974-020-01839-9)
Supplement: Supplementary file 1 — Additional file 1: Figure S1. Effects of IGF-1/PI3K signaling on the cytolysis of neurons induced by conditioned media. The concentration of PI3K was increased in cerebral cortical neurons (a) and cerebellar neurons (b) incubated with conditioned media of the OMS + NB group. The cytolysis of cerebral cortical neurons (c) and cerebellar neurons (d) incubated with conditioned media of the OMS + NB group was alleviated by exogenous IGF-1, which was suppressed by pretreatment with the PI3K inhibitor LY294002. ***p < 0.001, one-way ANOVA, n=20 (PBS, NB; IGF-1, NB), n=10 (PBS, OMS+NB; IGF-1 OMS+NB; DMSO, IGF-1, OMS+NB; LY294002, IGF-1, OMS+NB). [file 12974_2020_1839_MOESM1_ESM.docx]

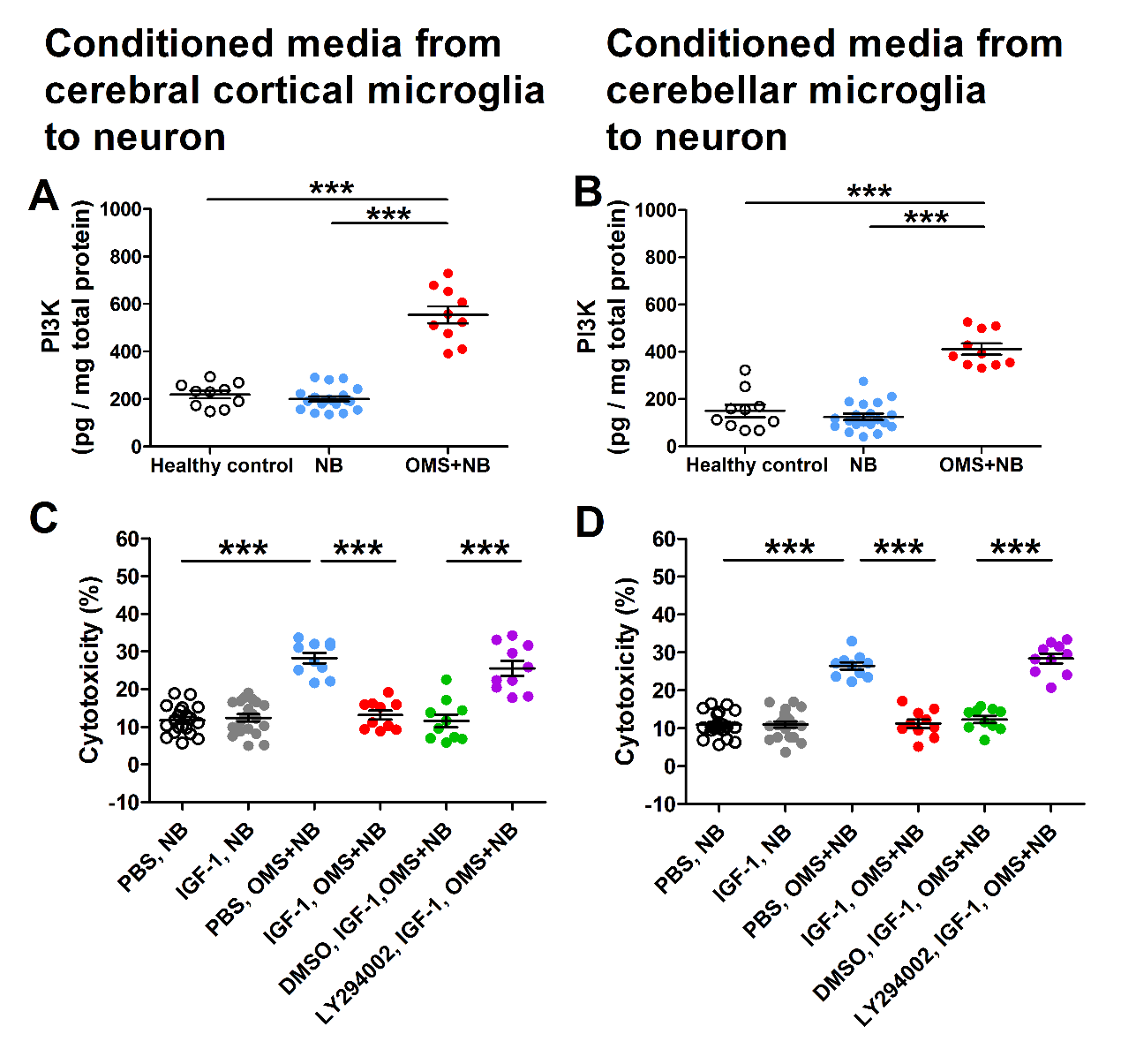


**Figure S1.** Effects of IGF-1/PI3K signaling on the cytolysis of neurons induced by conditioned media. The concentration of PI3K was increased in cerebral cortical neurons (**a**) and cerebellar neurons (**b**) incubated with conditioned media of the OMS + NB group. The cytolysis of cerebral cortical neurons (**c**) and cerebellar neurons (**d**) incubated with conditioned media of the OMS + NB group was alleviated by exogenous IGF-1, which was suppressed by pretreatment with the PI3K inhibitor LY294002. ^***^*p* < 0.001, one-way ANOVA, n=20 (PBS, NB; IGF-1, NB), n=10 (PBS, OMS+NB; IGF-1 OMS+NB; DMSO, IGF-1, OMS+NB; LY294002, IGF-1, OMS+NB).
